# Progressive Physiotherapy Rehabilitation Program for a Patient With Biceps Tenodesis Recovery: A Case Report

**DOI:** 10.7759/cureus.56085

**Published:** 2024-03-13

**Authors:** Ishwin Kaur B Bagga, Swapnil U Ramteke, Ashish Keoliya

**Affiliations:** 1 Department of Sports Physiotherapy, Ravi Nair Physiotherapy College, Datta Meghe Institute of Higher Education and Research, Wardha, IND

**Keywords:** dash score, case report, physical rehabilitation, constant-murley score, plyometric exercises, tecar therapy, low-level laser therapy, biceps tenodesis

## Abstract

The present case report investigates the effectiveness of a progressive physiotherapy rehabilitation program in aiding the recovery of a patient who underwent biceps tenodesis. It is a surgical procedure involving the reattachment or relocation of the biceps tendon to alleviate pain and enhance function in conditions, like tendinitis or tears. The rehabilitation program is specifically tailored to address the distinct challenges associated with biceps tenodesis recovery, focusing on gradual exercises aimed at improving strength, range of motion (ROM), and functional capacity. Through a comprehensive analysis, this case report seeks to offer insights into the potential advantages and obstacles of employing a specialized physiotherapy approach in the holistic care of individuals undergoing biceps tenodesis, contributing to the ongoing development of postoperative rehabilitation strategies.

## Introduction

Pathology involving the long head of the biceps brachii (LHBB) frequently leads to discomfort in the shoulder. Continuous pronation and supination, particularly during activities involving resistance or overhead motions, may result in tendon damage. Such damage could manifest as tendinosis or tears, displacement of the LHBB toward the medial side, traumatic ruptures of the LHBB, or instability associated with SLAP lesions [1]. The incidence of distal biceps tendon rupture is around 2.55 per 100,000 patient-years [1]. The incidence of LHBB rupture varies but tends to increase with age, particularly in individuals engaged in repetitive overhead activities or those with pre-existing shoulder issues. Biceps tenodesis, a surgical procedure to address such ruptures, has seen a rise in incidence due to its effectiveness in restoring function and alleviating pain, particularly in active individuals seeking to maintain shoulder strength and stability. Advances in surgical techniques have contributed to its popularity as a viable treatment option. Biceps tenodesis is advised for cases where over half of the tendon is torn, when the biceps tendon shifts toward the middle, or when there are tears in both the subscapularis and biceps leading to the latter's displacement. Alternatively, one could opt for either a straightforward surgical release or tenotomy [1,2].

Injuries to the long head of the biceps can sometimes be associated with issues in the rotator cuff, but they can also occur due to specific incidents, like trauma or injections. Damage to the biceps tendon is often a component of a broader degenerative condition affecting various structures around the shoulder, such as the rotator cuff, bursa, and possibly the acromioclavicular joint [3].

Transferencia electrica capacitiva resistiva (TECAR) therapy, a recent advanced therapy, is a non-invasive treatment and employs high-frequency currents to generate heat within tissues. This energy induces mechanical, chemical, biological, and thermal effects, enhancing blood flow management and promoting therapeutic benefits [4]. Low-level laser therapy (LLLT) lasers are referred to as ''low intensity'' or ''cool'' due to their minimal temperature variations. Many academics are skeptical about the effectiveness of LLLT due to its relatively low power output (milliwatts). LLLT can cause serious biological impacts [5,6,7].

TECAR therapy is renowned for its capacity to induce internal heat and holds significant therapeutic potential. Its extended electromagnetic wavelength enables profound tissue penetration, reaching muscles, tendons, and bones. With frequencies ranging from 300 kHz to 1 MHz, TECAR therapy is applicable even in acute phases, offering analgesic, anti-inflammatory, myorelaxant, tissue regeneration, and wound healing benefits. This versatile approach addresses various physiotherapy concerns effectively [8,9,10]. TECAR treatment (TT) is a form of thermotherapy that utilizes electrical currents to heat both surface and deep tissues. Its ability to affect blood circulation is seen as a key factor in facilitating tissue healing. TECAR employs capacitive and resistive energy transfer to achieve its therapeutic effects [11,12,13].

## Case presentation

Patient information

A 52-year-old male patient, a resident of Darwha, Yavatmal, India, a laborer by occupation, came to Acharya Vinobha Bhave Rural Hospital (AVBRH) on December 13, 2023, with complaints of pain, swelling, and weakness around his left arm. The patient gave a history of lifting heavy rocks, suddenly after which a click sound was heard from his left arm. Sudden pain and swelling were associated. The pain was sudden in onset, excruciating in nature, non-progressive, and aggravated by any movement of the arm. The patient visited a local practitioner where an MRI was done, and the patient was diagnosed with a tear of the long head of the biceps tendon on the left side. The patient was advised for surgery and was given medications. The patient came to AVBRH for biceps tenodesis and was operated on December 18, 2023 for biceps tenodesis. The patient was referred for physiotherapy, and a planned physical therapy rehabilitation protocol was designed for faster recovery of the patient. Physiotherapy aimed to improve the range of motion (ROM), increase strength, relieve pain, promote tissue healing, and reduce inflammation and edema.

Clinical findings

The patient's informed consent was taken. The patient exhibited an endomorphic physique and was conscious, cooperative, and well-oriented to time, place, and person. The patient had a reduced ROM and reduced strength. Table 1 shows the ROM assessment of the patient. Table 2 shows the manual muscle testing (MMT) assessment of the patient.

**Table 1:** Range of motion assessment of the patient

Joint	Pre-intervention (left)		Post week 4 intervention (left)	
	Active	Passive	Active	Passive
Shoulder: Flexion	0-100°	0-126°	0-132°	0-165°
Extension	0-18°	0-25°	0-30°	0-55°
Abduction	0-130°	0-138°	0-146°	0-158°
Medial rotation	0-30°	0-39°	0-48°	0-57°
Lateral rotation	0-20°	0-25°	0-28°	0-37°
Elbow: Flexion	0-112°	0-120°	0-135°	0-144°
Extension	112°-0	120°-0	135°-0	144°-0
Forearm: Pronation	0-40°	0-51°	0-70°	0-75°
Supination	0-34°	0-40°	0-70°	0-75°

**Table 2:** Manual muscle testing assessment of the patient

Joint	Pre-intervention (left)	Post week-4 intervention (left)
Shoulder: Flexion	2	3
Extension	2	3
Abduction	2	3
Medial rotation	2	3
Lateral rotation	2	3
Elbow: Flexion	2	3
Extension	2	3

Diagnostic assessment

**Figure 1:**
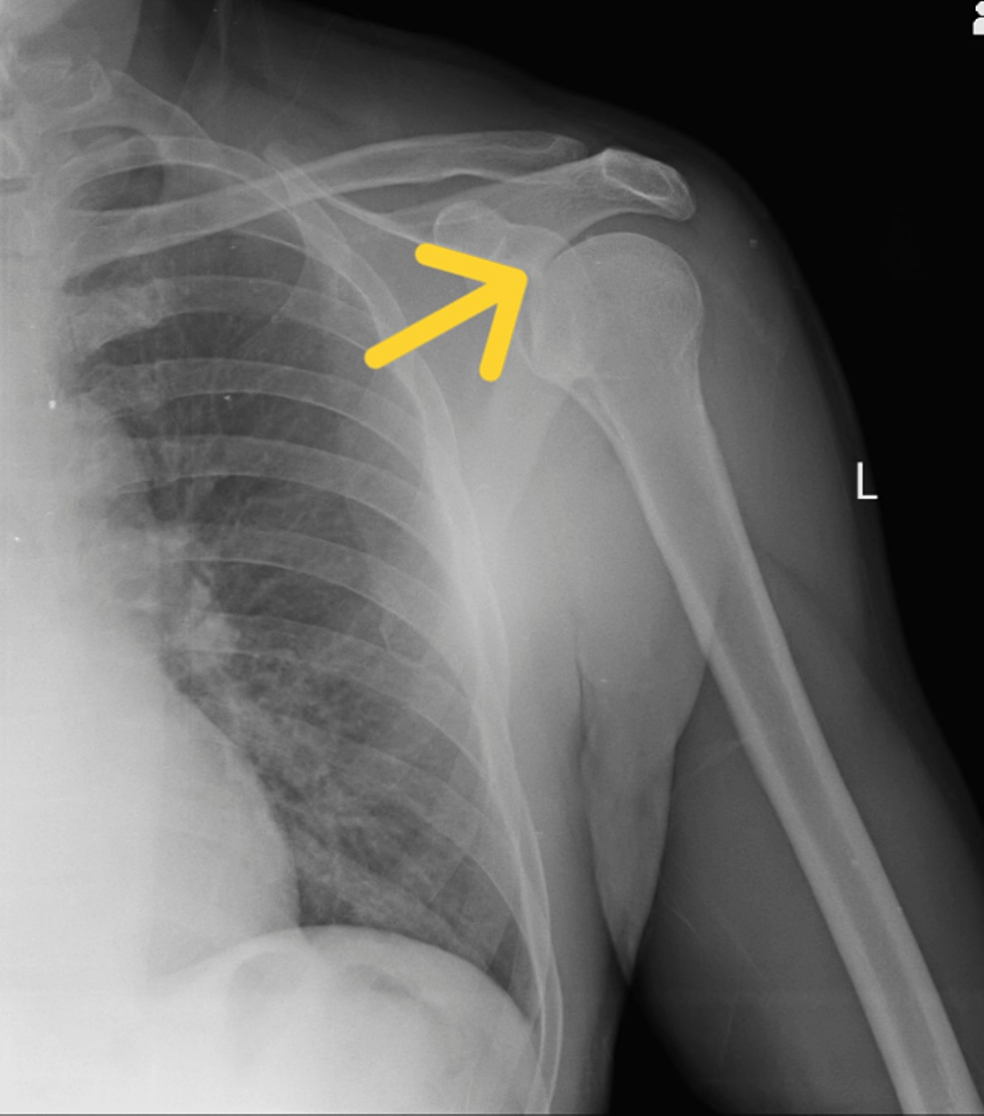
X-ray showing the anterior humerus dislocation The yellow arrow shows the site of dislocation.

**Figure 2:**
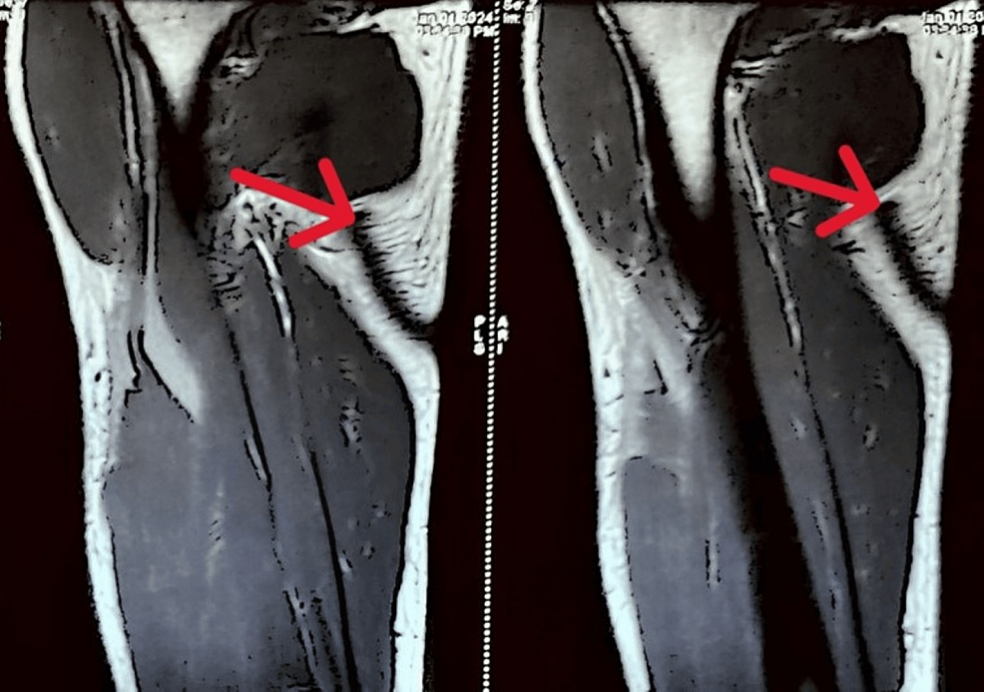
MRI showing the tear of the long head of the biceps tendon The red arrow shows the site of the tear.

The patient's X-ray revealed that the patient was having anterior humerus dislocation on the left side. The MRI findings showed that there was a tear in the long head of the biceps tendon on the left side. Figure 1 shows the anterior humerus dislocation X-ray. Figure 2 shows the MRI of the tear of the long head of the biceps.

Physiotherapy management

**Figure 3:**
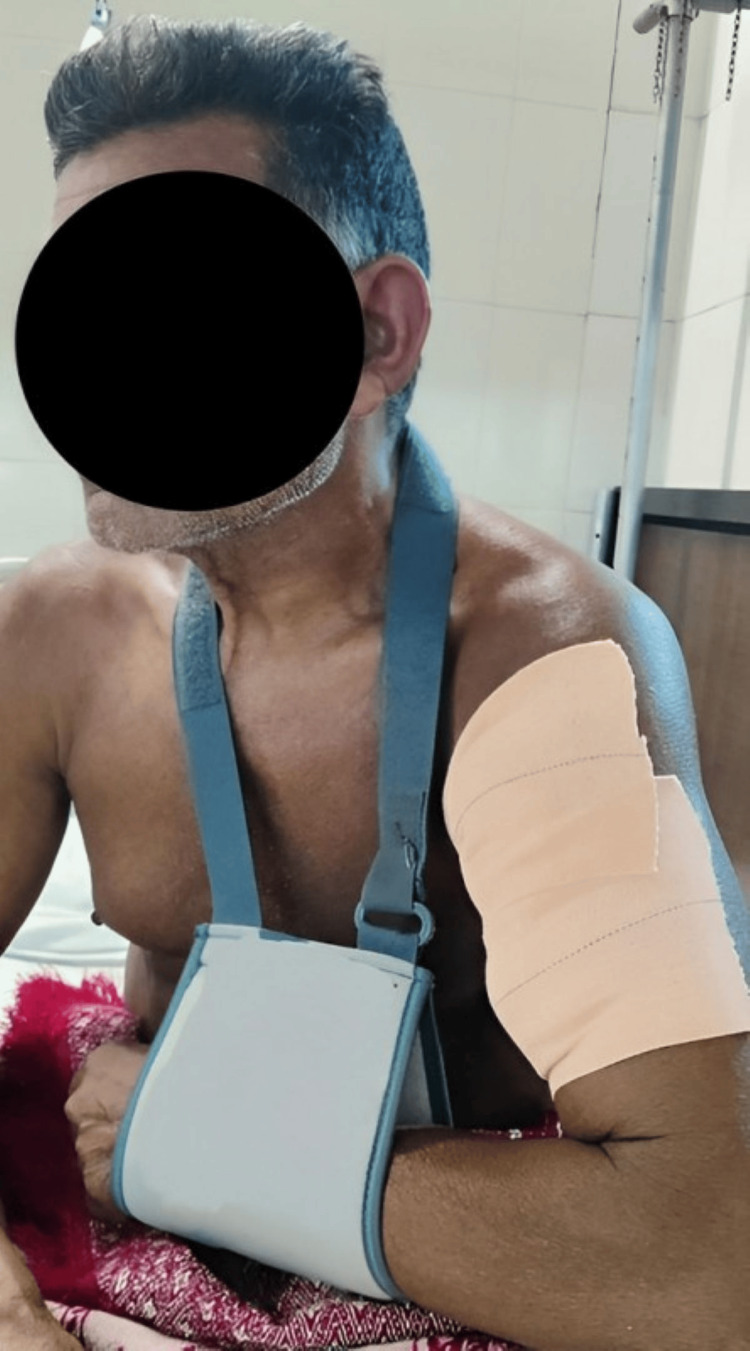
Use of a shoulder immobilizer (arm pouch) for support to the arm

**Figure 4:**
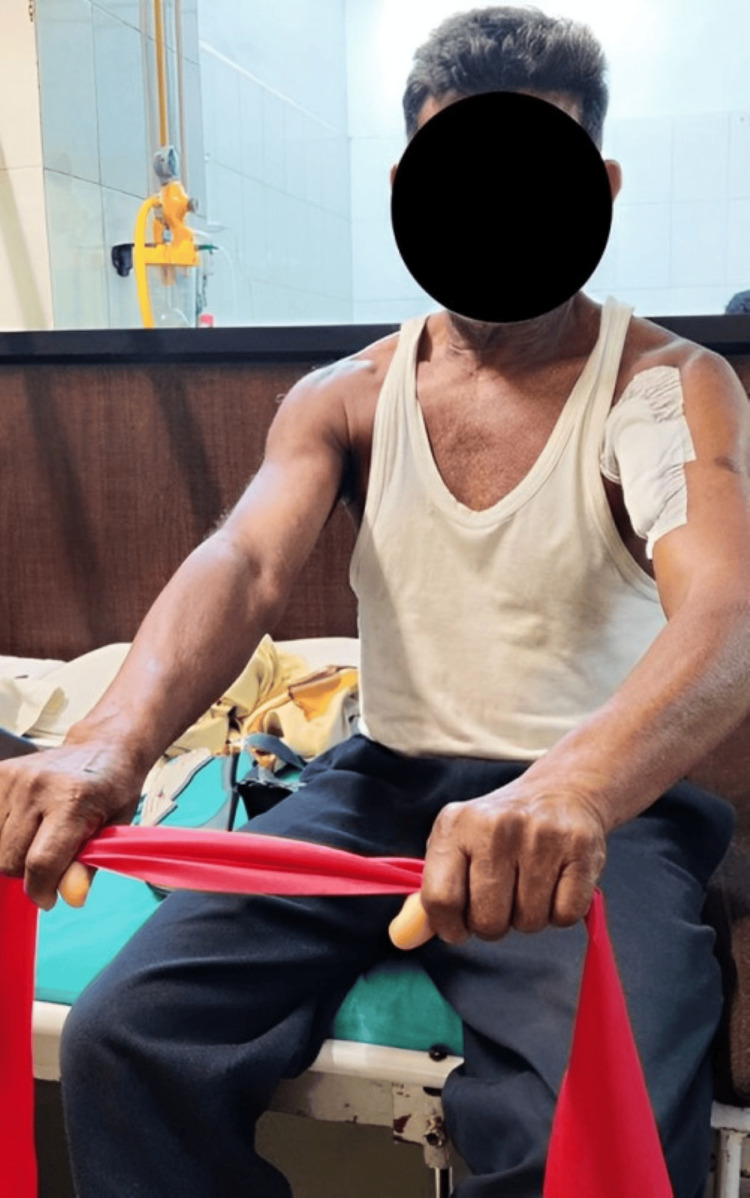
Patient performing strengthening exercise using theraband

**Figure 5:**
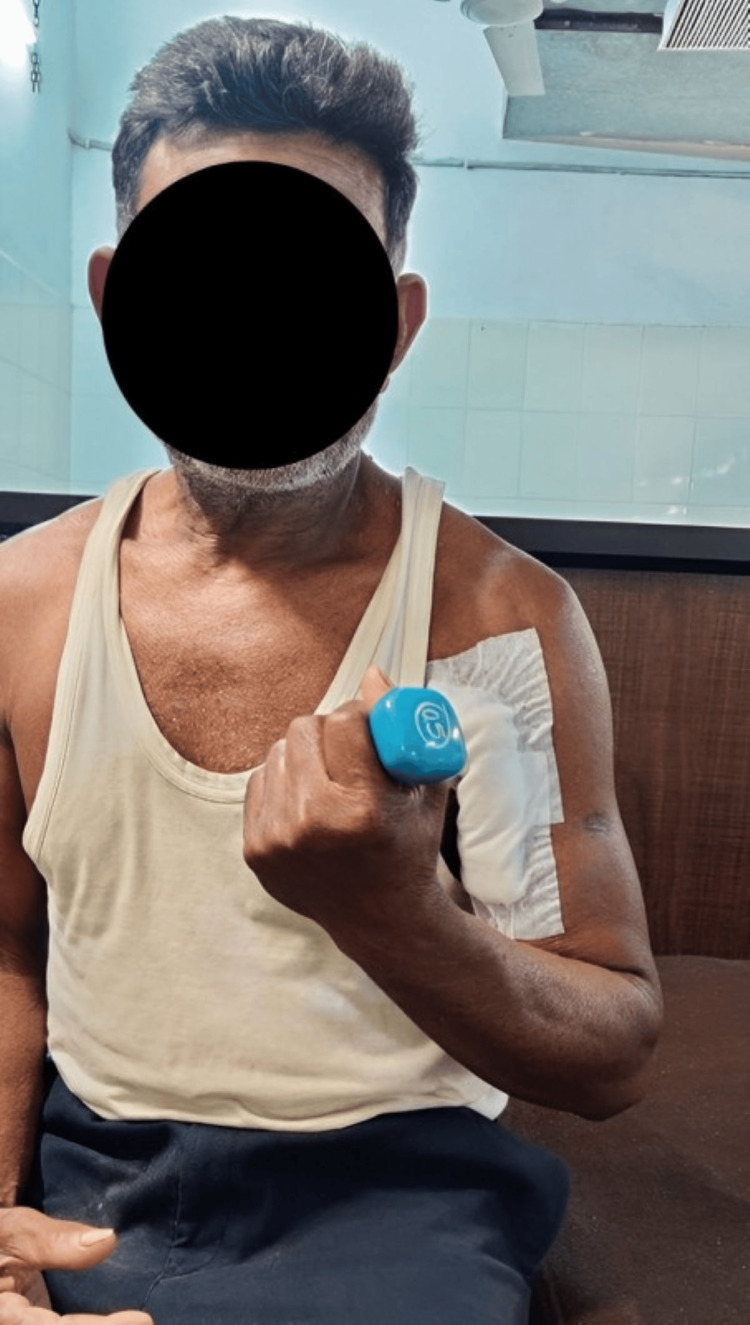
Strengthening exercises for shoulder flexors

Chief complaints of the patient were decreased ROM and reduced strength and inability to perform activities of daily living (ADLs), like eating, drinking water, combing hair, and dressing. A planned physiotherapy protocol was designed for the patient. Table 3 shows the planned physiotherapy program. Figure 3 shows the use of a shoulder immobilizer for support to the arm. Figure 4 shows the patient performing a strengthening exercise using a theraband. Figure 5 shows strengthening exercises for shoulder flexors.

**Table 3:** Planned rehabilitation protocol for the patient ADL: activity of daily living

Phase	Duration	Goals	Treatment regimen			Rationale
Immediate postoperative phase	Week 1	To control pain, edema, and inflammation. To promote tissue healing.		Reduce pain via cryotherapy for 10-15 mins [1].		Minimize postoperative complications and discomfort.
		To protect the surgical site.		Use the arm pouch for protection.		Prevent excessive stress on healing tissues.
		To initiate gentle ROM for shoulder flexors.		Initiate gentle ROM exercises for shoulder flexors.		Improve joint mobility.
Early mobility phase	Weeks 2-4	To optimize pain and edema. To promote tissue healing.		LASER- low-level laser therapy (energy density = 8J/cm^2 ^duration = 2 min 5 sec., frequency = 700 Hz) [14]		Reduce pain, inflammation, and edema and promote tissue healing.
		To restore normal shoulder mobility.		Gradual reduction in the use of an arm pouch. Early muscle activation.		Facilitate tissue healing.
		To strengthen gradually the rotator cuff muscles and elbow flexors and extensors.		Passive and active-assisted ROM exercises using a wand, gentle scapular bracing, shoulder shrugs, and other exercises. Elbow isometrics.		Prevent muscle atrophy.
Intermediate strengthening phase	Weeks 4-6	To increase rotator cuff and elbow flexors and extensor strength.	Progressive ROM - begin active ROM avoiding excessive stress on biceps [15].			Enhances joint stability and functioning.
			Passive stretching for biceps.			
			Isometric and isotonic strengthening to rotator cuff muscle.			
			Strengthening of supination action of biceps by supinated biceps curls with 10-sec hold 10-15 repetitions 3-5 sets [16].			
			Light resistance training for elbow flexors and extensors.			
			Initiate endurance training.			
			Progression of scapular exercises.			
Advanced strengthening phase	Weeks 6-8	To achieve full ROM, strength, and endurance.		Full ROM - focus on stretching and flexibility exercises.	Promote long-term joint health and function.	
				Progress to resisted ROM exercises.		
				Plyometric exercises (ball throwing) to improve shoulder dynamic stability.		
		To improve proprioception and coordination.		Progressive resistance training- 15-sec hold 10-15 repetitions 3-5 sets [16].	Restoration of ADLs.	
				Emphasize scapular stabilization while upper extremity movement.		
Maintenance and prevention phase	Weeks 8-12	To restore ADLs and to make the patient return to work.		Education on proper biomechanics and posture.	Education on ongoing self-management and self-care regimen.	
		To sustain strength and flexibility gains.		Continuation of exercises and progression in strengthening 20-sec hold 10-15 repetitions 3-5 sets [16].	Maintaining strength and ROM of the joint.	
				Home exercise program for maintenance and prevention of further injuries [17].	Avoiding re-occurrence of the condition.	

Outcome measures

The outcome measures used were the numerical pain rating scale (NPRS) to assess pain and the Constant-Murley score and Disabilities of the Arm, Shoulder, and Hand (DASH) to assess the severity of the affected ADLs. Table 4 shows the outcome measures assessment pre- and post-intervention.

**Table 4:** Outcome measures taken for the patient ADLs = Activities of daily living

Outcome measure	Pre-intervention	Post-intervention
Numerical pain rating scale	On rest: 5/10, on activity: 8/10 (severe pain while rest and doing ADLs)	On rest: 2/10, on activity: 4/10 (mild pain while rest and doing ADLs)
Constant-Murley score	Score: 64/100 (mediocre; patient had severe pain while doing ADLs)	Score: 88/100 (excellent; patient can do ADLs without pain)
Disabilities of the arm, shoulder, and hand (DASH)	Score: 85 (patient had severe disability in performing ADLs)	Score: 20 (patient is very mild disabled in performing ADLs)

## Discussion

Biceps tenodesis is an orthopedic surgery that can be done to repair tendons that connect the biceps muscle of the shoulder joint. In the above case report, we present the case of a 52-year-old male who had complaints of pain and swelling over the left arm, due to which ADLs were affected. Investigations via X-ray and MRI were done, which revealed a tear in the long head of the biceps muscle and were suggestive of bicipital tenodesis. The outcome measures used in the study are NPRS, Constant-Murley score, and DASH. To prevent future complications, like weakness, biceps tendon re-rupture, and nerve injury, it is very necessary to plan a good rehabilitation protocol. In the above study, various physiotherapy interventions, like strengthening of rotator cuff muscles and biceps, stretching, cryotherapy, ROM exercises, plyometrics for biceps and rotator cuff, and endurance training, were used.

Varacallo et al., in their study on biceps tendon dislocation and instability, found that physiotherapy regimens target the pathology of biceps tendon. It helps to strengthen the structures of the shoulder and contributes to a faster and more effective recovery [18]. Chen and Voloshin, in their review of treatment options for the long head of biceps injury, concluded that the best non-operative management and initial management for the long head of biceps rupture is physical therapy. He found that the use of cryotherapy and strengthening exercises are of vital importance [19]. Deutch et al. in their study noted that after biceps tenodesis, ranges that are mainly affected are flexion, supination, and pronation along with reduced strength. They found that physiotherapy can help improve ranges and strength effectively [20].

TECAR therapy, a recent advanced therapy, is a non-invasive treatment that employs high-frequency currents to generate heat within tissues. This energy induces mechanical, chemical, biological, and thermal effects, enhancing blood flow management and promoting therapeutic benefits [4]. In this case report, the patient was given strengthening and stretching of the biceps and rotator cuff, plyometric exercises, cryotherapy, functional training, and ROM exercises that showed a positive response in the patient and helped him in faster recovery, improving his overall general strength.

## Conclusions

The progressive physiotherapy rehabilitation used in biceps tenodesis recovery has been proven very effective for patients. This approach targets specific challenges related to the procedure while also improving overall strength, flexibility, and function. Patients experience a better ROM, muscle strength, and reduced pain, showing how well this method works. This case report highlights how crucial physiotherapy is for recovery after biceps tenodesis, showing its potential to improve quality of life after surgery.

TECAR therapy is a promising new treatment for conditions, like biceps tenodesis. However, it has not been studied in this report due to availability and cost issues. There has not been any research on using TECAR therapy specifically for biceps tenodesis, but it is known to boost circulation and help repair torn tendons, suggesting that it could be worth studying in the future.
